# Diagnosis and treatment of mast cell disorders: practical recommendations

**DOI:** 10.1590/1516-3180.2013.1314590

**Published:** 2013-08-01

**Authors:** Alex Freire Sandes, Raphael Salles Scortegagna Medeiros, Edgar Gil Rizzatti

**Affiliations:** I MD, PhD. Medical Consultant in Hematology, Division of Laboratory Medicine and Pathological Anatomy, Grupo Fleury, São Paulo, Brazil.; II MD. Medical Consultant in Pathological Anatomy, Division of Laboratory Medicine and Pathological Anatomy, Grupo Fleury, São Paulo, Brazil.

**Keywords:** Mastocytosis, Diagnosis, Anatomy, Flow cytometry, Therapeutics, Mastocitose, Diagnóstico, Anatomia, Citometria de fluxo, Terapêutica

## Abstract

**CONTEXT AND OBJECTIVE:**

The term mastocytosis covers a group of rare disorders characterized by neoplastic proliferation and accumulation of clonal mast cells in one or more organs. The aim of this study was to assess the principal elements for diagnosing and treating these disorders.

**DESIGN AND SETTING:**

Narrative review of the literature conducted at Grupo Fleury, São Paulo, Brazil.

**METHODS:**

This study reviewed the scientific papers published in the PubMed, Embase (Excerpta Medica Database), Lilacs (Literatura Latino-Americana e do Caribe em Ciências da Saúde) and Cochrane Library databases that were identified using the search term “mastocytosis.”

**RESULTS:**

The clinical presentation of mastocytosis is remarkably heterogeneous and ranges from skin lesions that may regress spontaneously to aggressive forms associated with organ failure and short survival. Currently, seven subtypes of mastocytosis are recognized through the World Health Organization classification system for hematopoietic tumors. These disorders are diagnosed based on clinical manifestations and on identification of neoplastic mast cells using morphological, immunophenotypic, genetic and molecular methods. Abnormal mast cells display atypical and frequently spindle-shaped morphology, and aberrant expression of the CD25 and CD2 antigens. Elevation of serum tryptase is a common finding in some subtypes, and more than 90% of the patients present the D816V *KIT* mutation in mast cells.

**CONCLUSION:**

Here, we described the most common signs and symptoms among patients with mastocytosis and suggested a practical approach for the diagnosis, classification and initial clinical treatment of mastocytosis.

## RESUMO

**CONTEXTO E OBJETIVO:**

O termo mastocitose abrange um grupo de raras doenças caracterizado por proliferação neoplásica e acúmulo de mastócitos clonais em um ou mais órgãos. O objetivo do presente estudo foi avaliar os principais elementos para o diagnóstico e tratamento dessas desordens.

**TIPO DE ESTUDO E LOCAL:**

Revisão narrativa da literatura realizada no Grupo Fleury, São Paulo, Brasil.

**MÉTODOS:**

O presente estudo revisou artigos científicos publicados nas bases de dados PubMed, Embase (Excerpta Medica Database), Lilacs (Literatura Latino-Americana e do Caribe em Ciências da Saúde) e Cochrane Library, que foram identificados com o termo de busca “mastocitose”.

**RESULTADOS:**

A apresentação clínica da mastocitose é marcadamente heterogênea, variando de lesões cutâneas que podem regredir espontaneamente, até formas agressivas associadas a falência de órgãos e curta sobrevida. Atualmente, sete subtipos de mastocitose são reconhecidos pela classificação de tumores hematopoéticos da Organização Mundial de Saúde; o diagnóstico é realizado com base nas manifestações clínicas e na identificação de mastócitos neoplásicos por métodos morfológicos, imunofenotípicos, genéticos e moleculares. Mastócitos anômalos apresentam morfologia atípica, frequentemente fusiforme, e expressão aberrante dos antígenos CD25 e CD2. Aumento de triptase sérica é um achado comum em alguns subtipos; e mais que 90% dos pacientes apresentam mastócitos com a mutação *KIT D816V*.

**CONCLUSÃO:**

No presente artigo, descrevemos os sintomas e sinais mais comuns em pacientes com mastocitose e sugerimos uma prática abordagem para o diagnóstico, classificação e tratamento clínico inicial.

## INTRODUCTION

Mastocytosis is currently defined as a heterogeneous group of disorders characterized by clonal expansion and accumulation of mast cells in one or more tissues, such as skin, bone marrow, liver, spleen, gastrointestinal tract and lymph nodes, among others.^1^ Clonal mast cells are detected in most cases and in all subtypes of the disease. These clonal mast cells are most often characterized by the presence of the D816V-activating *KIT* mutation; however, other classes of *KIT* mutations have been detected.^2^ Mastocytosis is considered to be one of the eight subcategories of myeloproliferative neoplasms, according to the 2008 World Health Organization classification system.^3^

There are two ages of peak onset for this disease: in the first decade of life and between the fourth and fifth decades of life.^4^^,^^5^ The skin is the organ most affected in this class of disorders; in fact, the skin is infiltrated in virtually all children and around 85% of adults with mastocytosis.^4^ The clinical presentation of mastocytosis is heterogeneous and ranges from disease limited to the skin (i.e. cutaneous mastocytosis) to cases with extracutaneous involvement (i.e. systemic mastocytosis). Cutaneous mastocytosis occurs mainly during infancy and childhood (i.e. most cases appear within the first year of life) and is commonly associated with spontaneous regression of skin lesions, whereas systemic mastocytosis is usually diagnosed in adults and ranges from indolent to aggressive forms of mastocytosis. The latter is associated with multiorgan failure and decreased survival.^3^

Mastocytosis is considered to be a rare disorder, but the true incidence and prevalence in the general population are unknown. In Brazil, it can reasonably be assumed that the diagnosis of mastocytosis is greatly underestimated, given the low clinical awareness of the disease and the existence of very few specialized diagnostic centers.

## OBJECTIVES

The aim of the present study was to review the main signs and symptoms of mastocytosis and suggest a practical approach for diagnosis, classification and initial management of patients with mast cell disorders.

## METHODS

We conducted a narrative review by means of a systematic literature search using the PubMed, Embase (Excerpta Medica Database), Lilacs (Literatura Latino-Americana e do Caribe em Ciências da Saúde ) and Cochrane Library databases to identify published scientific papers relating to the search term “mastocytosis.” Moreover, the papers identified needed to focus on human subjects (Table 1).

**Table 1.** Database search results

Database	Search	Results	
PubMed	Mastocytosis [Mesh]	2,747 papers	1,144 case reports 440 reviews 52 clinical trials 2 meta-analyses 1,109 others
Embase (Excerpta Medica Database)	Mastocytosis [Emtree]	2,701 papers	962 case reports 554 reviews 168 clinical trials 1,017 others
Lilacs (Literatura Latino-Americana e do Caribe em Ciências da Saúde)	Mastocytosis	81 papers	45 case reports 36 others
Cochrane Library	Mastocytosis	18 papers	18 clinical trials

## RESULTS

### Clinical manifestations

The clinical symptoms of mastocytosis are caused by acute and chronic release of intracytoplasmic mast cell mediators (i.e. histamines, tryptase, prostaglandins and leukotrienes) and tissue infiltration by neoplastic mast cells. Common symptoms include pruritus (i.e. generalized or restricted to cutaneous lesions), skin redness and swelling, sweating, palpitations, thoracic pain and headache.^1^^,^^5^^,^^6^ Recurrent abdominal pain and diarrhea are present in a significant number of cases and may evolve to severe malabsorption, thereby leading to weight loss and hypoalbuminemia. Osteoporosis can also be found in a small number of cases.

Anaphylactic reactions associated with vascular collapse and risk of death occur in approximately 20% of adults and 6% of children, and these extreme symptoms are more frequent in males.^4^ In some cases, the allergic triggering factor is not identified, whereas in others, a history of medication use (e.g. aspirin, nonsteroidal anti-inflammatory drugs and iodinated radiocontrast agents) or insect bites has been linked to the onset of symptoms. In mast cell leukemia, repeated severe episodes of massive release of mast cell mediators are typical, and these patients must be treated in intensive care units. In clinical subtypes other than mast cell leukemia, however, there may not be any direct relationship between mast cell mass, mast cell release and symptoms.^7^

In many patients, the first sign of the disease is a characteristic maculopapular rash known as urticaria pigmentosa. The symptoms associated with these lesions are exacerbated with friction (Darier’s sign).^7^^,^^8^ In aggressive forms, the massive tissue infiltration can lead to signs and symptoms secondary to the existence of hepatic or bone marrow infiltration. These additional signs and symptoms include abdominal pain, portal hypertension and ascites (i.e. relating to hepatic infiltration) and pancytopenia (i.e. relating to bone marrow infiltration).^9^

### Diagnosis and classification of mastocytosis

The diagnosis of mastocytosis is based on identification of neoplastic mast cells by means of morphological, immunophenotypic and/or genetic methods.^10^ The World Health Organization classification system defines the following seven subtypes of the disease: cutaneous mastocytosis, indolent systemic mastocytosis, systemic mastocytosis associated with other clonal hematological non-mast cell lineage diseases, aggressive systemic mastocytosis, mast cell leukemia, mast cell sarcoma and extracutaneous mastocytoma.^3^

Cutaneous mastocytosis is characterized by abnormal mast cell infiltration in the dermis and no evidence of systemic involvement. The following three variants of cutaneous mastocytosis have been described: urticaria pigmentosa, diffuse cutaneous mastocytosis and solitary mastocytosis of the skin. Urticaria pigmentosa is the most common presentation in children and represents 70-90% of cases of mastocytosis in this population. The most affected areas include the trunk and extremities, whereas the palms, soles, scalp, and face are less frequently compromised.^7^

**Chart 1.**
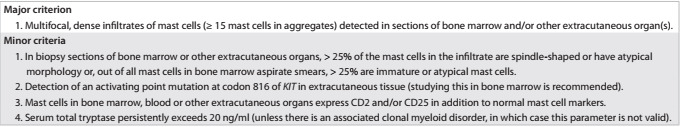
Diagnostic criteria for systemic mastocytosis according to the World Health Organization (WHO) classification

**Figure 1.**
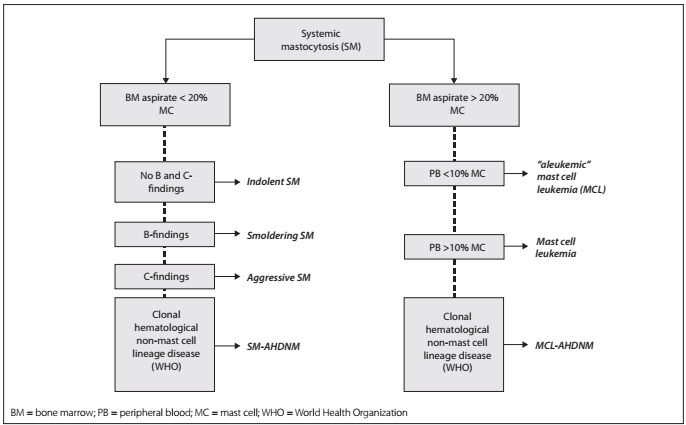
Algorithm for classification of systemic mastocytosis (SM).

Diagnosing systemic mastocytosis requires identification of major and minor criteria in the patient’s case history (Chart 1). More specifically, the diagnosis of systemic mastocytosis is confirmed when one major plus one minor criterion or three minor criteria are fulfilled. The subclassification of systemic mastocytosis is defined according to the tumor burden of mast cells (B-findings), the clinical aggressiveness of the disease (C-findings) and the involvement of hematopoietic lineages other than mast cells (Table 2 and Figure 1). In this last situation, both systemic mastocytosis and the components of systemic mastocytosis associated with other clonal hematological non-mast cell lineage diseases are present, according to the World Health Organization’s criteria.^3^

**Table 2.** B and C-findings in mastocytosis

	B-findings (Increased MC burden)	C-findings (Impaired organ function)
Bone marrow	- BM MC > 30% - Serum tryptase > 200 ng/ml - Hypercellular and dysplastic BM, without MDS criteria	PB cytopenia: Neutrophils < 1 x 10^9^/l Hemoglobin < 10 g/dl Platelets < 100 x 10^9^/l
Spleen and liver	Hepatomegaly or splenomegaly in absence of functional impairment	- Hepatomegaly with ascites, abnormal liver function or portal hypertension - Palpable splenomegaly with hypersplenism
Gastrointestinal tract	-	Intestinal malabsorption, associated with hypoalbuminemia and weight loss
Skeletal lesions	-	Osteolytic lesions, osteoporosis and pathological fractures associated with local MC infiltration

BM = bone marrow; MC = mast cell; PB = peripheral blood; MDS = myelodysplastic syndrome.

Mast cell sarcoma and extracutaneous mastocytoma are extremely rare and characterized by localized mast cell tumors with no evidence of systemic mastocytosis. Mast cell sarcoma has been reported as occurring in the uterus,^11^ intestine,^12^ bone^13^ and skin.^14^ Moreover, it typically presents a destructive growth pattern and high-grade cytology. On the other hand, extracutaneous mastocytoma cases have been reported to involve the lung and to present low-grade cytology and a non-destructive growth pattern.^3^

Recently, two new categories previously not included in the World Health Organization classification system were described: clonal mast cell activation disorders characterized by recurrent episodes of idiopathic anaphylaxis, absence of skin lesions and the presence of only one or two minor World Health Organization criteria for systemic mastocytosis;^15^^,^^16^^,^^17^ and well differentiated systemic mastocytosis characterized by mast cells with normal morphology, absence of CD25 and CD2 expression and detection of *KIT* mutations other than D816V. Well differentiated systemic mastocytosis may be responsive to treatment with imatinib (e.g. mutations of *KIT-*F522C).^18^^,^^19^

Another subtype previously not included in the World Health Organization classification system is myelomastocytic leukemia, an even rarer disease described in patients with advanced stages of myeloid neoplasms (e.g. refractory anemia with excess of blasts and acute myeloid leukemia). This manifestation is generally associated with a high number of atypical mast cells that do not meet the criteria for systemic mastocytosis.^20^^,^^21^ Myelomastocytic leukemia typically involves an increased number of myeloblasts (i.e. > 5%) and metachromatic blasts (i.e. > 10%) in the peripheral blood and/or bone marrow and lacks other features of mast cell disorders, such as mast cell infiltrates, expression of CD25 and CD2, or *KIT-*D816V mutation.

#### Bone marrow aspirate

Normal and reactive mast cells are round or oval and small to medium sized cells that present a central round nucleus, condensed chromatin without a nucleolus, abundant cytoplasm (i.e. low N/C ratio) and numerous metachromatic cytoplasmic granules, as identified via Romanowsky-based staining methods.

**Figure 2.**
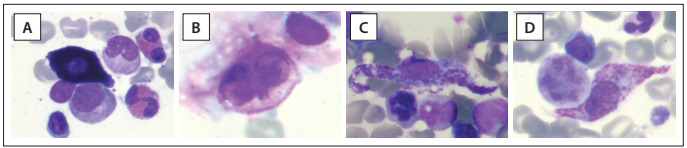
Bone marrow aspirates from a healthy individual (panel A) and from systemic mastocytosis cases (panels B to D). Panel B demonstrates an atypical mast cell type II, with a bilobed nucleus; panels C and D show spindle-shaped atypical mast cells type I, with eccentric oval nuclei and cytoplasmic projections.

The cytomorphology of neoplastic mast cells have been classified into the following three subtypes:^22^ (1) metachromatic blasts with a high N/C ratio, fine nuclear chromatin, prominent nucleoli and few metachromatic granules; (2) atypical mast cells type I, which are spindle-shaped cells with oval nuclei in an eccentric position, elongated cytoplasmic projections and hypogranular cytoplasm; and (3) atypical mast cells type II (promastocytes)*,* which exhibit bi- to multilobed nuclei associated with mature morphology (i.e. condensed chromatin and low N/C ratio) or immature morphology (i.e. fine chromatin and high N/C ratio) (Figure 2). Moreover, these morphological features of mast cells have been correlated with different clinical presentations of mastocytosis. Atypical mast cells type I are more commonly found in systemic mastocytosis cases with an indolent course, whereas atypical mast cells type II and metachromatic blasts are more frequently found in mast cell leukemia cases, which are commonly associated with poor outcome and shorter survival time.

The percentage of mast cells in the bone marrow is decisive for the final diagnosis of systemic mastocytosis at advanced stages, and an increase in the number of mast cells to ≥ 5% suggests an unfavorable prognosis. In patients with mast cell leukemia, the number of mast cells in the bone marrow smear is ≥ 20%. The differential count should be performed in areas far from bone spicules, since mast cells tend to be present in higher numbers near the spicules. It should be noted that the limit of 20% applies only to bone marrow smears but not to histological sections.^22^ In addition, a cytomorphological analysis contributes towards detection of eosinophilia, myelodysplasia and additional morphological features that might suggest coexistence with another hematological malignancy.^10^

#### Bone marrow histopathology

Presence of multifocal, dense infiltrates of mast cells (i.e. > 15 mast cells in aggregates) in bone marrow trephine biopsy sections and/or other extracutaneous organs is a major criterion for systemic mastocytosis, according to the World Health Organization classification.^1^^,^^3^ Documentation of bone marrow involvement accompanying systemic mastocytosis is often established by examination of a bone marrow trephine biopsy specimen. Indeed, histological sections demonstrate multifocal clusters or cohesive aggregates/infiltrates of mast cells mainly in the form of peritrabecular and intertrabecular distribution. The presence of mast cells can be confirmed by Giemsa staining, which enables observation of their main morphological features, i.e. a blue to purple-colored pattern and the typical cytoplasmic granules. However, these features can also be observed under reactive mast cell conditions and in cases of myelomastocytic leukemia.

**Figure 3.**
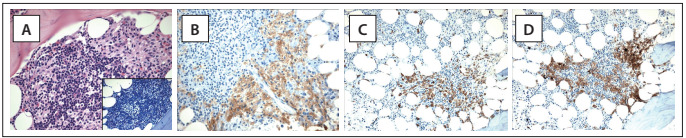
(A) Hematoxylin-eosin-stained photomicrograph of bone marrow showing a cluster of packed fusiform cells with eosinophilic cytoplasm and some degree of atypia, in which there are large nuclei with clumped chromatin and nucleoli. These cells are permeated by small reactive lymphocytes. The mast cell nature is highlighted on a Giemsa-stained slide (in inset detail), showing as bluish hypogranulated cytoplasm. (B) Immunolabeled stain for CD 117 confirming the mast cell nature of the cells, which also aberrantly express tryptase (C) and CD 25 (D). These findings confirm the diagnosis of bone marrow mastocytosis.

Cytomorphological features can help to differentiate normal/reactive mast cells from neoplastic mast cells. In tissue sections stained with hematoxylin and eosin, normal/reactive mast cells are usually loosely scattered throughout the sample and display round to oval nuclei with clumped chromatin, a low N/C ratio and an absent or indistinct nucleolus. The cytoplasm is abundant and usually filled with small, faintly visible granules that are best highlighted by Giemsa staining. Dense aggregates of mast cells are only exceptionally detected in reactive states or in patients treated with stem cell factor. For the diagnosis of systemic mastocytosis, atypical features of mast cell must be observed, such as a compact cluster of both spindle-shaped and round mast cells in varying numbers intermingled with lymphocytes, eosinophils, histiocytes and fibroblasts, all of which are more frequently seen in indolent systemic mastocytosis (Figure 3). Less often, the clusters are more monomorphic and mainly composed of spindle-shaped mast cells that abut or stream along the bony trabeculae. Significant reticulin fibrosis and thickening of the adjacent bone trabeculae are also frequently observed.

A diffuse pattern of infiltration is the predominant pattern in aggressive systemic mastocytosis and mast cell leukemia.^23^ In these patients, systemic mastocytosis can be diagnosed without additional tests, since the presence of one major criterion (i.e. aggregates of mast cells) and one minor criterion (i.e. abnormal morphology) match the World Health Organization’s diagnostic criteria.

This is not the case in patients with tryptase-positive round cell infiltrates of mast cells (TROCI), in which further tests are necessary for diagnosis (e.g. staining for CD117, CD25 and CD34 by means of flow cytometry), given that basophils and myeloblasts can also express low levels of tryptase.^24^ Coexpression of CD25 and CD117 in tryptase-positive round cell infiltrate cells is highly suggestive of systemic mastocytosis.

In addition, detection of a single infiltrate of a mast cell or presence of mast cell aggregates with less than 15 cells should be considered to be a minor diagnostic criterion.^1^ It should be noted that absence of mast cell infiltrates in bone marrow biopsies has been reported in around 20-30% of indolent systemic mastocytosis cases,^17^^,^^25^ thereby suggesting that lower sensitivity towards the World Health Organization major criterion is needed in diagnosing systemic mastocytosis at the initial stages of the disease.^10^

The most specific methods for identifying immature or atypical mast cells in tissue sections are based on immunohistochemical staining for tryptase and CD117.^23^^,^^26^ These neoplastic mast cells aberrantly express CD2 and CD25, which are the best markers for the definitive diagnosis of systemic mastocytosis.^10^

Finally, careful inspection of the hematopoietic characteristics other than the mast cells in the bone marrow is of crucial importance. Often, the unaffected bone marrow is seemingly typical with normal distribution of fat cells and hematopoietic precursors. Such cases usually either belong to indolent systemic mastocytosis with involvement of the skin and bone marrow or represent scenarios of isolated mastocytosis of the bone marrow. In other cases, the bone marrow may be extremely hypercellular due to proliferation of cells of non-mast cell lineage. These findings may be reactive (i.e. myeloid hyperplasia) or may indicate a coexisting hematopoietic neoplasm. Lymphoproliferative diseases are less frequently identified in this setting. Clinicians must pay special attention to increased cellularity of the bone marrow and disturbed maturation of hematopoietic cells, because these patterns may be associated with an unfavorable outcome or with a smoldering variant of systemic mastocytosis, even if the criteria for a coexisting myeloid neoplasm are not fulfilled.

#### Multiparameter flow cytometry

Immunophenotyping by means of flow cytometry provides relevant information for diagnosis, classification and monitoring of hematological malignancies.^27^^,^^28^^,^^29^^,^^30^^,^^31^ Normal and reactive mast cells in the bone marrow present high forward and sideward light scatter characteristics and are promptly detected through strong expression of CD117.^32^ The normal mast cell phenotype is characterized by expression of CD45, CD63, CD203c, FcRIe and cytoplasmic total tryptase (CyB12); and by absence of CD2, CD25, CD123 and CD34 antigens.^33^^,^^34^ HLA-DR is usually negative, but may also be partially expressed in a small fraction of normal individuals.

Mast cells in systemic mastocytosis patients frequently present aberrant antigenic expression, thereby allowing a means of clear differentiation from normal mast cells with diagnostic sensitivity higher than 98% and specificity of 100%.^32^ Thus, multiparameter flow cytometry is highly informative in this setting and is currently considered to be the gold standard for identification of aberrant mast cells.^10^

**Figure 4.**
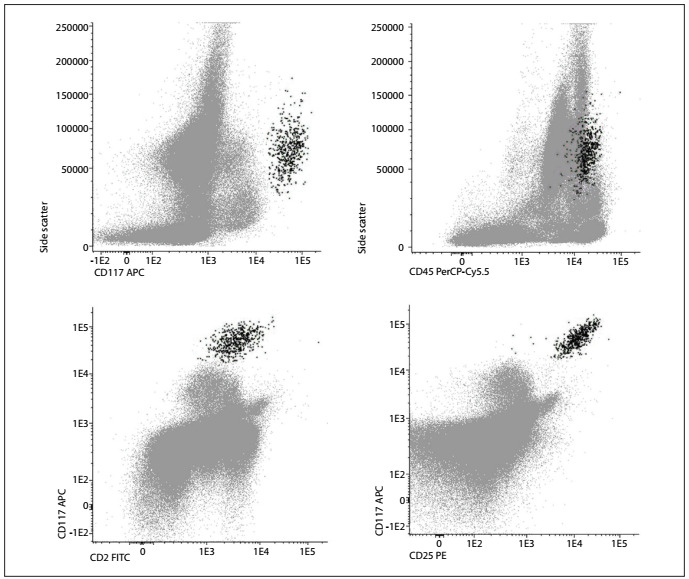
Representative bivariate dot plots illustrating mast cells (black) identified by means of flow cytometry based on CD117 and CD45 expression, with anomalous expression of CD2 and CD25, in a case of systemic mastocytosis.

Neoplastic mast cells usually demonstrate aberrant expression of CD25, with or without CD2, and abnormal expression of these markers is considered to be a minor diagnostic criterion in the World Health Organization’s classification system^10^^,^^32^^,^^33^^,^^34^^,^^35^^,^^36^^,^^37^ (Chart 1, Figure 4). Other abnormal phenotypic profiles that have been described include aberrant expression of CD123; overexpression of CD203c, CD63, CD69 or CD45; and low expression of CyB12.^34^^,^^38^

It was recently shown that systemic mastocytosis is phenotypically heterogeneous and presents three different maturation-associated immunophenotypic profiles that correlate with molecular subtypes of the disease and have prognostic relevance.^33^^,^^36^ Patients with indolent systemic mastocytosis and clonal mast cell activation disorders have an immunophenotypic profile similar to that observed in activated mast cells (i.e. overexpression of CD63, CD69 and CD203c), in addition to high forward and sideward light scatter characteristics, positivity to CD25 and high expression of CD2. In contrast, mast cells from well differentiated systemic mastocytosis cases show normal expression of activation markers, lack of both CD25 and CD2, a phenotype similar to that of mature resting mast cells and high expression of CD117 and FcRIe, as the main features. Finally, in cases of mastocytosis that are typically associated with unfavorable outcomes (i.e. aggressive systemic mastocytosis and mast cell leukemia), the mast cells exhibit aberrant expression of CD25 and usually do not express CD2. This phenotype has been associated with low light scatter values (i.e. forward and sideward light scatter, but especially low sideward light scatter) and low expression of CD117 and FcRIe, thereby reflecting an immature phenotype. Expression of CD30 is also related to distinct subtypes of mastocytosis, i.e. it is strongly positive in cases of aggressive systemic mastocytosis and negative or dimly expressed in cases of indolent systemic mastocytosis.^37^^,^^39^

#### Serum tryptase

Serum tryptase levels are elevated in most patients with systemic mastocytosis, and detection of serum tryptase levels higher than 20 ng/ml is considered to be a minor diagnostic criterion in the World Health Organization’s classification system.^1^^,^^5^^,^^40^ Tryptase levels tend to be higher in patients with a high mast cell burden; patients with aggressive systemic mastocytosis generally present tryptase levels > 200 ng/ml, especially as the disease progresses.^41^ However, tryptase levels are also elevated in a significant proportion of cases of acute myeloid leukemia, chronic myeloid leukemia and myelodysplastic syndromes, and should not be considered to be a diagnostic criterion for patients with suspected myeloid neoplasms associated with mastocytosis.^3^ Serum tryptase concentrations generally decrease after treatment of aggressive systemic mastocytosis and mast cell leukemia, thus making this a suitable marker for evaluating the response to cytoreductive drugs.^1^

#### c-KIT mutation and molecular studies

The *KIT-*D816V mutation is considered to be a minor diagnostic criterion according to the World Health Organization’s classification system. Indeed, it is detected in more than 90% of cases of mastocytosis and, as the number of pathological cells in the sample increases, the likelihood of detecting the *KIT* mutation also increases.^2^^,^^42^ The sensitivity of *KIT* mutation detection can be increased by enriching the mast cells in the sample by fluorescence-activated cell sorting and use of highly sensitive polymerase chain reaction techniques.^1^^,^^10^

In suspected systemic mastocytosis cases, the analysis should be performed using bone marrow samples collected in ethylenediaminetetraacetic acid, and both non-fractionated and mononuclear bone marrow cells can be examined for the *KIT* mutation. Peripheral blood should not be used as an alternative to bone marrow, since the mutation is not found in the peripheral blood of most patients with indolent systemic mastocytosis.^1^ Detection of the *KIT* mutation in the dermis is indicative of mastocytosis in the skin, but it is not diagnostically indicative of systemic involvement.

In rare cases of systemic mastocytosis, no *KIT-*D816V mutation is detected. In patients with small-sized mast cell infiltrates, a negative result must be interpreted with caution due to the small number of mast cells analyzed. Nevertheless, in aggressive systemic mastocytosis and suspected well differentiated systemic mastocytosis cases, demonstration of absence of D816V is clinically important since cases with wild type-*KIT* and certain types of *KIT* mutations other than D816V may respond to imatinib therapy.^19^ If no mutation at codon 816 is detected, sequencing of *KIT* should be considered.^1^ Moreover, in female patients without the *KIT* mutation, the pattern of chromosome X inactivation can be assessed by means of the human-androgen receptor-a gene (HUMARA) assay, in order to evaluate mast cell clonality.^25^

In the presence of peripheral blood eosinophilia (> 1500 cells/ml), investigation of the *CHIC-2* deletion and *FIP1L1-PDGFRA* rearrangement by means of fluorescence *in situ* hybridization or the reverse transcriptase polymerase chain reaction is indicated.^1^^,^^5^^,^^40^^,^^43^ Moreover, detection of translocations in regions containing chromosome bands 5q31-5q33 via conventional cytogenetic analysis generally enables identification of cases associated with the *PDGFRB* rearrangement.^43^^,^^44^ Cases that have either a *PDGFRA* or *PDGFRB* mutation associated with mast cell hyperplasia should be properly classified as “myeloid neoplasms with *PDGFRA* or *PDGFRB* rearrangements,” according to the World Health Organization’s classification system.^3^

### Treatment

**Chart 2.**
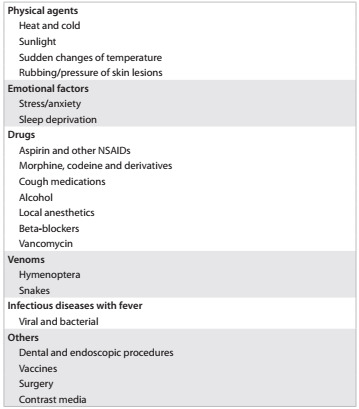
Triggering agents in mastocytosis

Treatment of mastocytosis is based on the specific clinical presentation and should take the following principles into account:^1^^,^^4^^,^^40^^,^^45^ (i) patients should receive detailed information about the disease and specific information concerning avoidance of agents and situations that trigger mast cell degranulation (Chart 2); (ii) treatment of symptoms associated with acute and chronic release of mast cell mediators should be part of the therapeutic regimen; and (iii) cytoreductive treatment should be restricted to cases involving advanced forms of the disease.

Patients with cutaneous mastocytosis and indolent forms of mastocytosis (i.e. indolent systemic mastocytosis and smoldering mastocytosis) must be treated symptomatically, i.e. with topical and systemic drugs that act on the release of mast cell mediators, such as histamine receptor antagonists (H1 and H2), sodium cromoglycate, and glucocorticoids (Oxford evidence level 1b). It is important to emphasize that most patients should not receive treatment to decrease the mast cell burden.^1^^,^^4^^,^^7^^,^^40^^,^^43^^,^^45^

Aggressive forms of mastocytosis (C-findings) should be treated with cytoreductive drugs; more specifically, interferon-alpha (i.e. 3 to 5 million units/m^2^/day) and cladribine (i.e. 5 mg/m^2^/day x 5 days q 4-6 weeks; 3 cycles) are the most widely used agents (Oxford evidence level 2b).^46^^,^^47^^,^^48^^,^^49^^,^^50^ Cases with the *KIT-*D816V mutation are resistant to treatment with imatinib, and this drug should be reserved for patients with *KIT* mutations other than the D816V mutation (Oxford evidence level 2b).^51^^,^^52^ Despite the fact that second-generation tyrosine kinase inhibitors (e.g. dasatinib) have shown *in vitro* efficacy against mast cells with the D816V mutation,^53^ recent clinical studies have reported only modest activity in D816V-positive systemic mastocytosis cases (Oxford evidence level 2b).^54^ New tyrosine kinase inhibitors, such as midostaurin (PKC412), are currently being evaluated and appear promising as potential therapeutic agents for systemic mastocytosis cases.^51^^,^^55^ Bisphosphonates should be used in cases with marked osteopenia and osteoporosis (Oxford evidence level 2b).^56^

Despite the limited evidence supporting hematopoietic stem cell transplantation, this treatment is an alternative approach and may induce remission in selected cases with advanced systemic mastocytosis (i.e. aggressive systemic mastocytosis and mast cell leukemia) (Oxford evidence level 3b).^57^^,^^58^ Valent et al. recommend that a debulking phase consisting of polychemotherapy or repeated cycles of cladribine should be included before performing the hematopoietic stem cell transplantation.^45^

In contrast to C-findings, B-findings are not indicative of the presence of aggressive forms of the disease and should not lead to treatment decisions unless the symptoms progress (i.e. convert to C-findings). The standard message is “B: Borderline, Benign, Be careful - wait and watch whether progression occurs; C: Consider cytoreduction.”^1^

Recently, hypomethylating agents (i.e. decitabine and 5-azacytidine) were tested in vitro and showed proapoptotic and growth-inhibitory effects on cultured neoplastic mast cells (Oxford evidence level 5).^59^ However, clinical trials are required to confirm the clinical efficacy of these compounds.

## CONCLUSIONS

Mastocytosis refers to a heterogeneous group of disorders that are characterized by accumulation of neoplastic mast cells in tissues. These disorders are diagnosed and classified based on clinical (i.e. B and C-findings), morphological, immunophenotypic (i.e. aberrant expression of CD25 and CD2) and molecular (i.e. *KITD816V* mutation) criteria. Correct subtype identification is essential for treatment and management of these disorders.
